# Progress in Pediatrics

**Published:** 1901-05-11

**Authors:** 


### Progress in Pediatrics.

(*Continued from page* 84.)

**The Congenital Stridor of Infants.**—The causation of this condition has been further investigated by Drs. John Thomson and Logan Turner.[6] They point out that the symptom has been ascribed by different authors to posticus paralysis, to adductor spasm from adenoids or other irritation, to an enlarged thymus, and to a congenital malformation of the upper aperture of the larynx. By means of a series of experiments on the cadaver the authors have shown that the epiglottis can be drawn down and curved inwards to close the upper aperture of the larynx in a manner which closely simulates that seen during life in congenital stridor cases, and that further this alteration in shape tends to persist if repeated several times. Arguing from these experiments the authors suggest that there exists in this affection, primarily, a disturbance of the co-ordination of the respiratory movements from delayed cerebral development, and, secondarily, an alteration in the shape of the upper aperture of the larynx as the result of the irregular breathing. They hold, therefore, that there is no congenital deformity in these cases, but that the anatomical changes are developed in the very early days of life.

**Splenomegalic Cirrhosis of the Liver.** — Dr. Frederick Taylor[7] relates a case of this affection, and discusses the nature of the lesion very fully. Under the above name there is now recognised a special disease which is characterised clinically by an enlarged cirrhotic liver, a very much enlarged spleen, jaundice, stunted growth, clubbing of the fingers, and sometimes pigmentation. This affection is to be distinguished from alcoholic cirrhosis, which may be regarded as due to local poisoning, and is rather to be regarded as due to some general infection, *e.g.* scarlatina or pneumonia. It is to be noted that the splenomegaly is not dependent on portal obstruction, but on the contrary is frequently an early change and one much more pronounced than the enlargement of the liver. Real obstructive results, such as ascites, are not usually present until nearly the end of the illness. Some French writers regard the important points in a case to be the relations, in time, of the hepatic and splenic lesions respectively, and classify the cases as follows:—(1) *Splenomegalic hypertrophic biliary cirrhosis*, when spleen and liver are equally and simultaneously affected; (2) *Meta-splenomegalic hypertrophic biliary cirrhosis*, when the spleen is markedly affected before the liver, secondary hepatitis; and (3) *Presplenomegalic hypertrophic biliary cirrhosis*, when the liver is markedly affected before the spleen, secondary splenitis. In some cases the disease would appear to manifest itself first by gastro-intestinal symptoms, in others by hepatic symptoms, jaundice, and enlargement of the liver, and in others by splenic symptoms, pain, and enlargement of the spleen. Histological examination of the liver shows that the distribution of the fibrous tissue is not exclusively multilobular or unilobular. The stunted growth and the clubbing of the fingers are at present quite unexplained. The disease is not a common one, but probably more cases will be published soon, and are much to be desired, as the whole condition is still shrouded in mystery.

**Hernia in Children.**—This subject was discussed at the annual meeting of the American Medical Association[8] last year. Dr. A. J. Ochsner gave as the chief predisposing causes the following—namely, the anatomy of the formation and descent of the testes, an unduly long mesentery, inherited local and systemic weakness, and flabbiness of the muscular tissues. The exciting factors are intra-abdominal pressure due to intestinal disturbance, flatulence, distension, constipation, urinary obstruction in phimosis, and obstinate cough or violent crying. Many hernias heal before the child's sixth year, when statistics show 1 to exist in 21 patients; from that to the thirteenth year the tendency to recovery is still present, so that the ratio is 1 in 77; and even later, up to adult life, recovery may occur. It has been shown that only 5 per cent. of all adult cases date their history back to infancy and childhood. In treatment the chief points are to keep the sac empty and to remove the cause, especially intestinal disorder, constipation, and phimosis. If the child be put into a bed with the foot raised, and all abdominal disturbance prevented, improvement will promptly begin in many cases. In other cases a truss is required night and day to flatten and close the canal. Operative measures should be reserved for patients over four years old, or for cases of irreducible, incarcerated, or strangulated hernia. Dr. de Garmo said that 95 per cent. of all cases are spontaneously cured by the fifth year, and that under ordinary conditions this should be the earliest period for operating. Confinement in bed was bad for the growing child, and a good truss which allowed of out-of-door exercise was to be preferred.

**Therapeutic Notes.**—Cacodylate of soda has been employed by Dr. Rocaz[9] in children with good results, more especially in the case of anæmia in infants, and in early tuberculosis. He administers it in aqueous solution by the mouth in doses varying from 1 to 3 milligrammes, according to the age of the child. Before commencing, it is advisable to ascertain that the kidneys are healthy, and in the course of treatment it is well to allow intervals of a few days frequently so as to avoid a possible cumulative effect. Dr. Levy[10] finds that in the treatment of chorea, the best way to administer arsenic is along with sodium chloride and mixed with butter. The mixture of sodium chloride and arsenic is triturated with 10 grains of fresh butter, and spread on bread, a form of administration which children like. It should be given at meal times, never on an empty stomach, and two doses per diem will usually suffice.

[6] Brit. Med. Jour., Dec. 1, 1900. [7] Guy's Hosp. Rep., Vol. LIV. [8] Med. News, June 23, 1900. [9] La Semaine Med., Oct. 31, 1900. [10] Epit. Brit. Med. Jour., Aug. 4, 1900.

